# Supplementary material for: Encoding extracellular modification of artificial cell membranes using engineered self-translocating proteins
Source: Nat Commun. 2024 Oct 30;15:9363. doi: 10.1038/s41467-024-53783-4 (PMC11526174; doi:10.1038/s41467-024-53783-4)
Supplement: Supplementary file 1 — Supplementary Information [file 41467_2024_53783_MOESM1_ESM.pdf]

## Supplementary Fig. 1: Leakage assays.

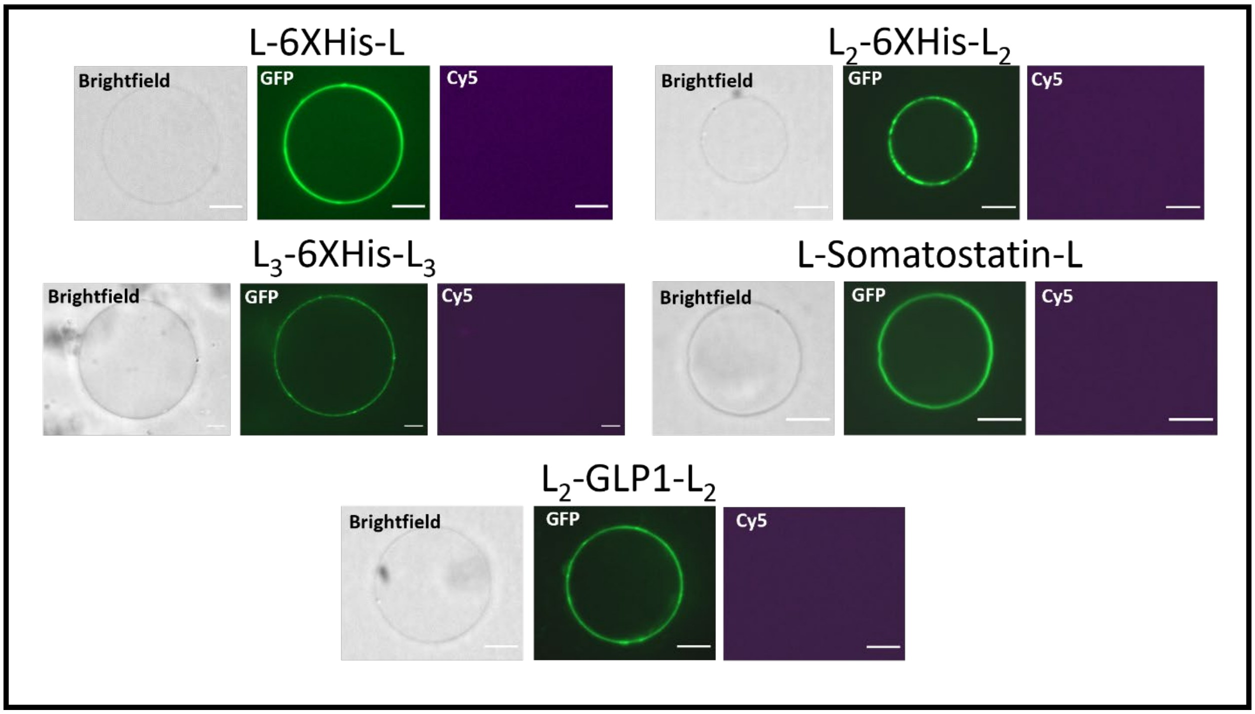


GUVs encapsulating Cy5 were formed through the inverse emulsion method with a lipid composition of 60 mol% DOPC and 40 mol% cholesterol. Addition of a GFP- αHL fusion protein leads to leakage of Cy5 from the GUVs , showing that αHL with the respective insert in the loop can assemble into functional pores. Shown are representative GUVs after 90 min of incubation with GFP- αHL fusion protein at RT. Leakage assays were replicated 3 times. Scale bar: 10 µm

**Supplementary Fig. 2: Cysteines in αHL with L-Somatostatin-L insert form a disulfide bond.**

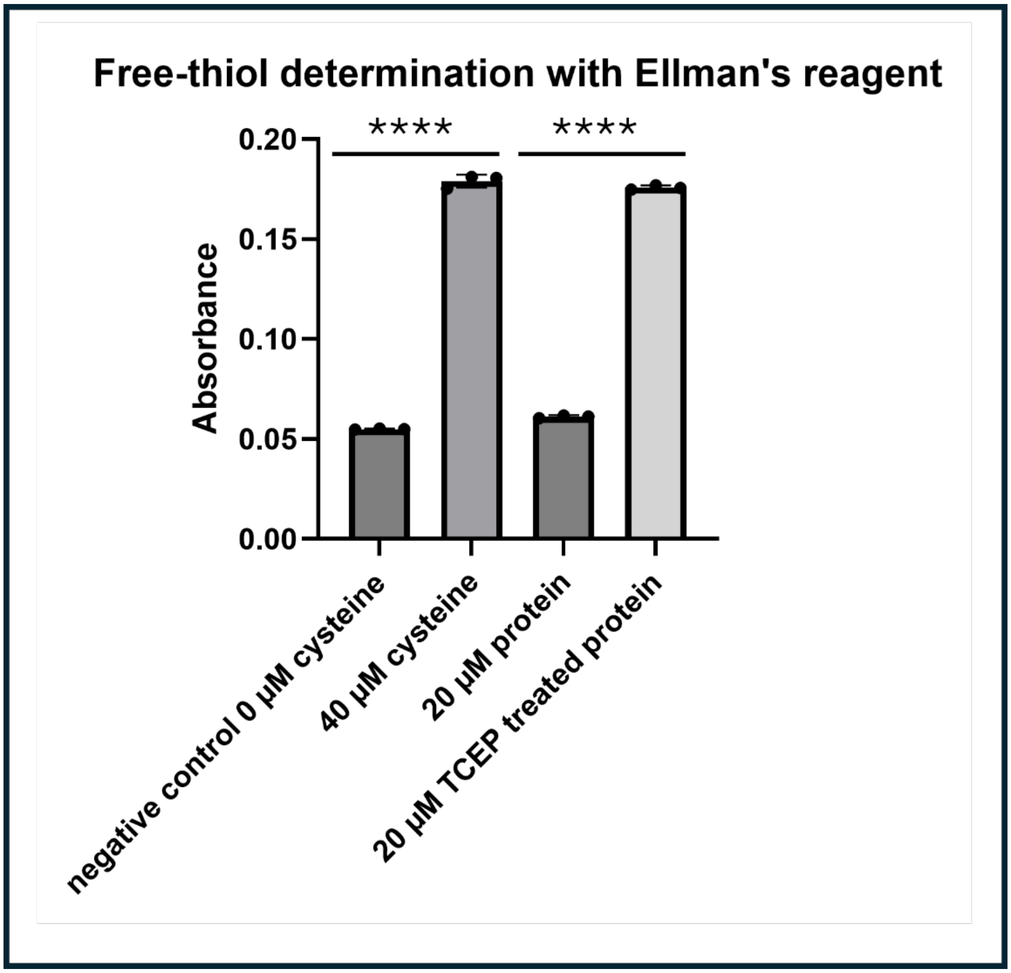


The concentration of free thiols in αHL with the L-somatostatin-L insert was determined using Ellman's reagent, following a previously published procedure[1]: A solution of 20 µM αHL with the L-somatostatin-L insert gives an absorbance value comparable to that of a negative control after treatment with Ellman's reagent. Reduction of the disulfide bond present in the protein and subsequent removal of the reducing agent makes the protein reactive to Ellman's reagent. Since the protein contains two free cysteines after reduction, 20 µM protein give a similar absorbance value to a 40 µM cysteine reference sample. Samples were measured as triplicates and the sample-mean is reported. Error bars represent standard deviation. Statistical significance was analyzed using an independent t-test (two-tailed). P-values are $<0.0001$ for both comparisons. Source data are provided as a Source Data file.

## Supplementary Fig. 3: Influence of translocated insert on leakage rate induced by αHL pores.

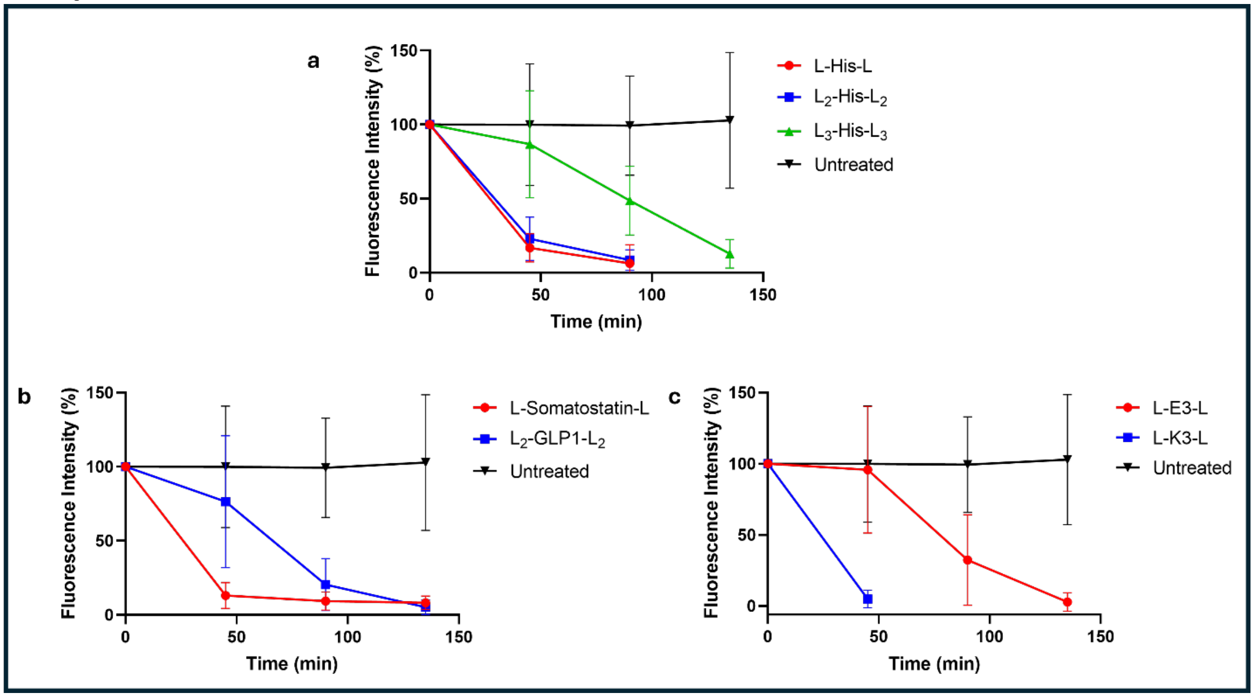


Cy5 encapsulating GUVs were treated with 1 µM of αHL-GFP containing the indicated insert and fluorescence of the GUVs was measured over time. Every 45 minutes, a population of GUVs (n>14) was imaged and analyzed for Cy5 fluorescence. Mean fluorescence intensity at different timepoints was calculated for each αHL mutant and for untreated control GUVs. Fluorescence at time point 0 was set to 100% to enable easier comparison of different inserts. Error bars represent standard deviation of the GUV population. **a**, Comparison of the 3 His-tag insert αHL: Increasing the length of the glycine serine linker decreases leakage rate. **b**, The same trend can be observed when comparing αHL with the somatostatin and the GLP1 insert. L-somatostatin-L αHL forms induces leakage faster than αHL containing the larger GLP1 insert. **c**, Apart from size, charge of the insert appears to also influence rate of leakage. While the K3 and E3 insert are of the same length, the positively charged K3 insert leads to leakage much faster than the negatively charged E3 insert. Electrostatic interactions between the negatively charged amino acids at the mouth of the pore and the negatively charged Cy5 dye, may contribute to the reduced leakage rate. An untreated negative control shows no leakage over the measured time period. Data was collected from n>14 GUVs and analyzed for statistical significance using an independent t-test (two-tailed): For each treatment group, statistical significance was tested for the first time point against the last time point. For the untreated group p = 0.9043. For all other groups p<0.0001. Source data are provided as a Source Data file.

While the leakage data shown in this figure can be used as a baseline for the design of new inserts, the data does not represent a quantitative measurement of small molecule transport across the respective pores. The leakage data presented is influenced by various additional variables such as the binding affinity of the monomer to the GUV membrane, as well as subsequent prepore assembly and prepore insertion.

## Supplementary Fig. 4: Negative controls for antibody assay.

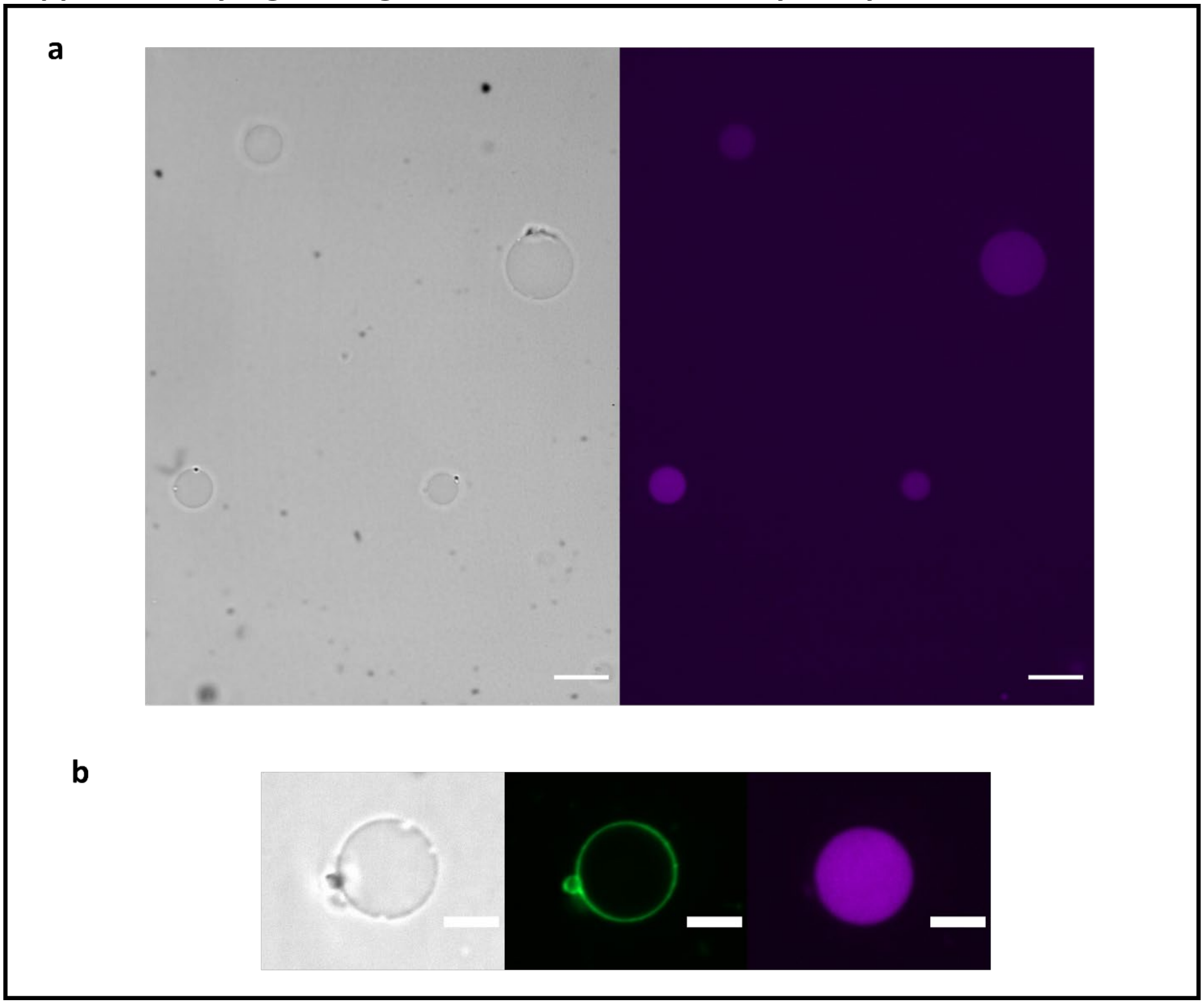


**a**, GUVs encapsulating Cy5 anti-6XHis-tag antibody show no membrane localization of antibody without αHL treatment after 2 h. Scale bar: 25 µm **b**, GUVs encapsulating Cy5 anti-6XHis-tag antibody show no membrane localization of Cy5-antibody after 4 h treatment with αHL with $L_2$-GLP1-$L_2$ insert. Scale bar: 10 µm. Shown are representative GUVs. Experiments were replicated twice.

**Supplementary Fig. 5: αHL with $L_2$-GLP1-$L_2$ insert expressed inside GUVs translocates GLP1 peptide to the outer membrane side.**

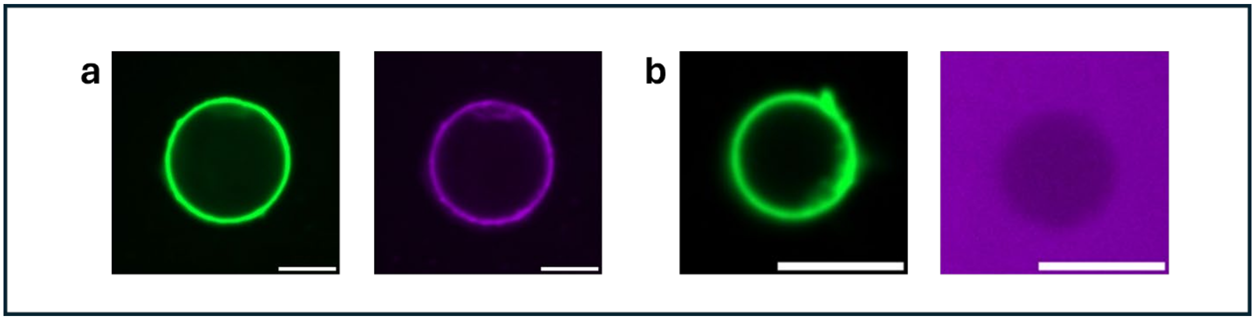


αHL with the $L_2$-GLP1-$L_2$ insert with a C-terminal GFP fusion was expressed inside GUVs using the PURExpress® system. GUVs encapsulating the PURExpress® in vitro expression system were formed as described in the methods section. After 2 hours of protein expression, Cy5-labeled anti-GLP1 or anti-Somatostatin (negative control) antibody was added externally at a dilution of 1:100. GUVs were incubated with the antibody for 1 h at RT before imaging. Externally added antibody clearly binds to the membrane, indicating that expression of αHL inside GUVs leads to translocation of the insert to the outer membrane side where it is accessible for antibody binding. **a**, Cy5-anti-GLP1 antibody binds the translocated insert and localizes to the GUV membrane. **b**, Cy5-anti-somatostatin antibody does not localize to the membrane showing that there is no unspecific binding to the GUV membrane. Shown are representative GUVs from two independent experiments.

**Supplementary Fig. 6: Quantification of antibody insert binding after translocation.**

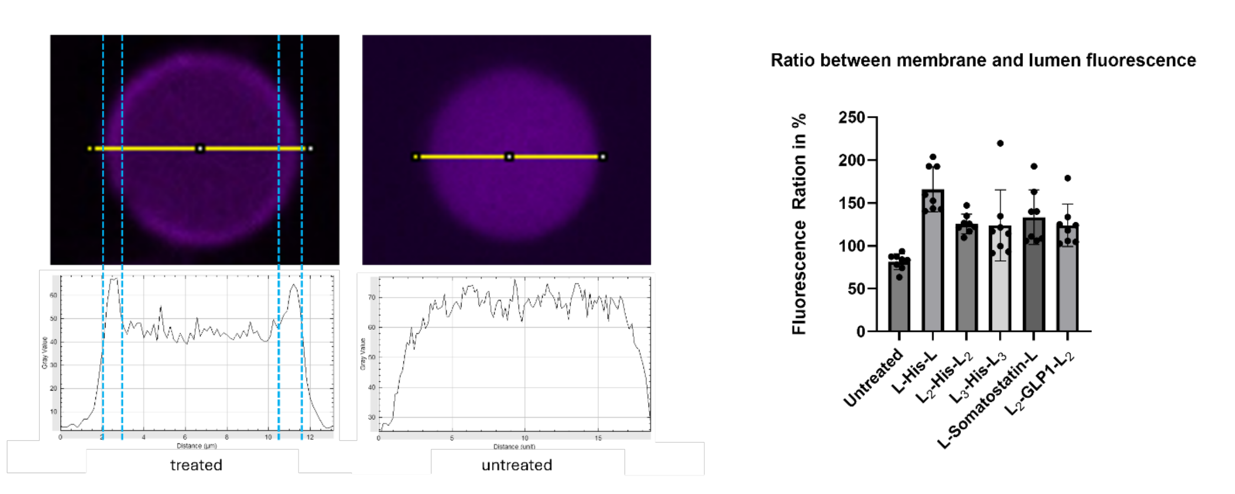


Quantitative analysis of the antibody binding assay as described in Fig. 3. **a**, Representative GUVs (n=8) were analyzed in ImageJ. Translocation of the peptide insert across the GUV membrane leads to localization of the Cy5 labeled antibody to the membrane and subsequently to increased fluorescence at the membrane. For each GUV, a fluorescence plot profile across the whole GUV was created. The generated fluorescence values were used to calculate a mean membrane fluorescence and a mean lumen fluorescence. The ratio of mean membrane fluorescence to lumen fluorescence is plotted in **b**. Untreated GUVs show a mean fluorescence ratio of 82%, whereas treatment with hemolysin constructs leads to a fluorescence ratio increase of at least 50%. Data (n = 8 vesicles per treatment group) was analyzed using an independent t-test (two-tailed). Statistical significance was tested for each treatment group against the untreated group. $p < 0.0001$ for L-His-L; $p < 0.0001$ for $L_2$-His-$L_2$; $p = 0.0135$ for $L_3$-His-$L_3$; $p = 0.0006$ for L-Somatostatin-L; $p = 0.0005$ for $L_2$-GLP1-$L_2$. Source data are provided as a Source Data file.

**Supplementary Fig. 7: Cryo-EM 2D class averages of αHL with $L_2$-GLP1-$L_2$ insert.**

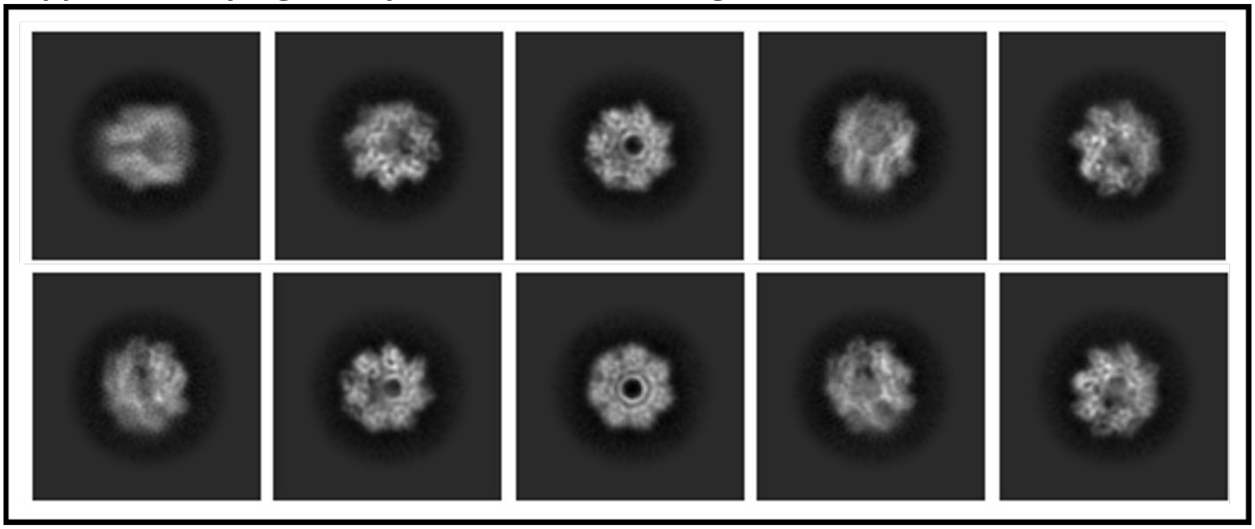

αHL monomer with the $L_2$-GLP1-$L_2$ insert was treated with sodium deoxycholate (6.25 mM) to induce pore formation.[2,3] The pore solution was diluted to 1 mM sodium deoxycholate and used for the preparation of cryo-EM grids. 2D class-averages were generated from 36,740 particles collected from 5357 exposures. These 2D class-averages further confirm that peptide inserts in the loop region do not significantly alter the 3D structure of the αHL pore. Due to the high flexibility of the GGGGSGGGGS linkers, it is not possible to visualize the insert in the loop through cryo-EM.

## Supplementary Fig. 8: Leakage assay for αHL with K3 and E3 insert.

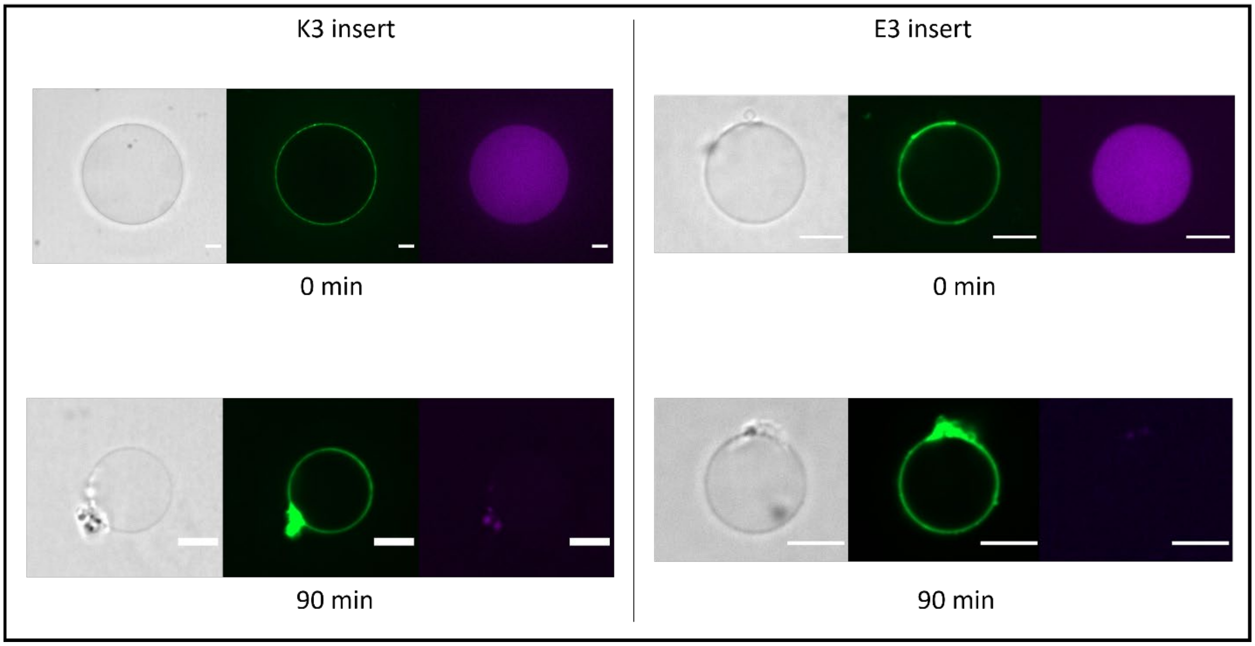


GUVs encapsulating Cy5 were formed through the inverse emulsion method with a lipid composition of 60 mol% DOPC and 40 mol% cholesterol. Addition of a GFP- αHL fusion protein with the K3 insert or E3 insert leads to leakage of Cy5 from the GUVs over time, showing that αHL with the K3 insert or E3 can assemble into functional pores. Shown are representative GUVs right after protein addition and after 90 min of incubation at RT. Leakage assays for αHL with K3 and E3 inserts were performed under low salt conditions. Leakage assays were replicated independently 3 times. Scale bar: 10 µm

**Supplementary Fig. 9: Sufficient protein expression is necessary for tissue formation.**

a

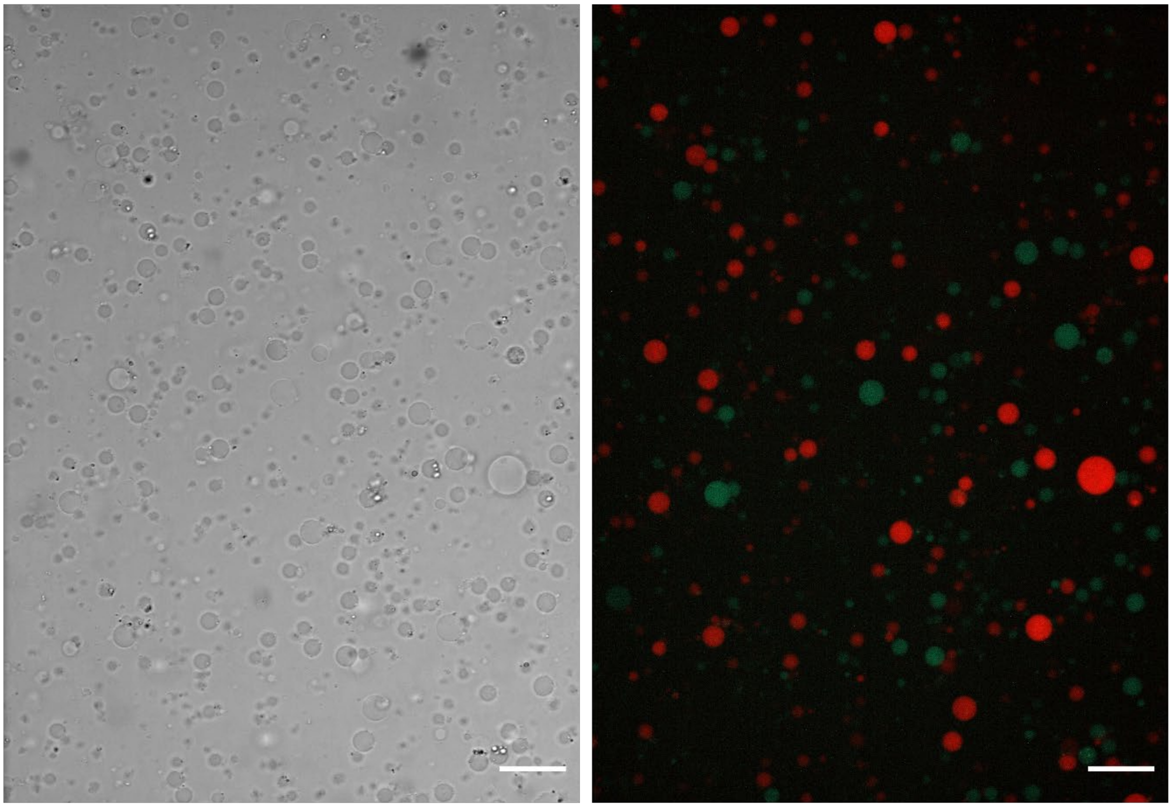

**a**, GUVs expressing αHL with either the K3 or the E3 insert were formed through the inverse emulsion method with a lipid composition of 60 mol% DOPC and 40 mol% cholesterol. The GUVs encapsulate the PURExpress system with a plasmid for expression of the αHL protein. For GUVs expressing αHL with the E3 insert, CFP was added to enable fluorescence imaging while for GUVs expressing αHL with the K3 insert mCherry was co-encapsulated. After incubation for 20 min at 37 °C no aggregation could be observed. Aggregation requires protein expression for at least one hour. Shown is a representative image from 3 independent experiments. Scale bar: 25 µm

## Supplementary Fig. 10: GUVs expressing αHL with K3 insert do not self-aggregate.

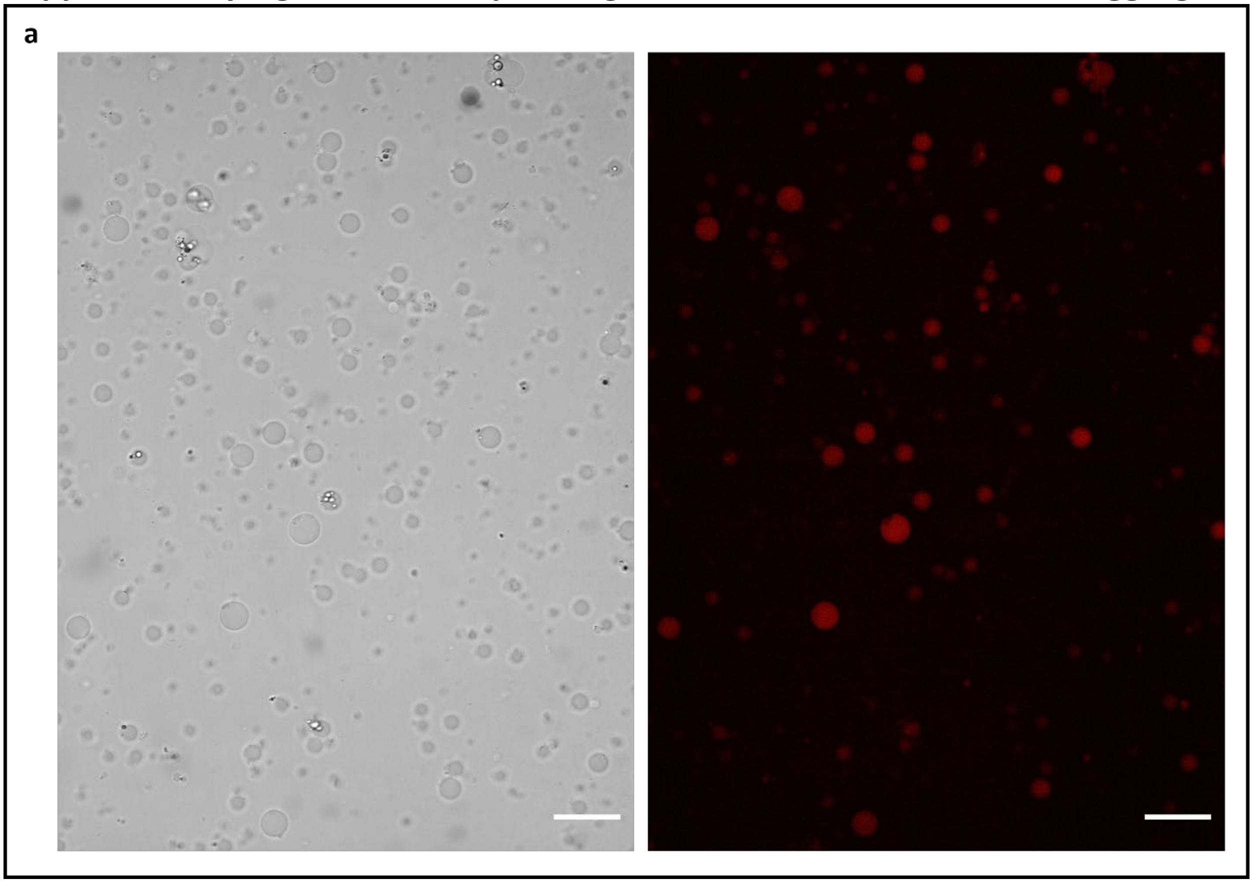


**a**, GUVs expressing αHL with the K3 insert were formed through the inverse emulsion method with a lipid composition of 60 mol% DOPC and 40 mol% cholesterol. The GUVs encapsulate the PURExpress system with a plasmid for expression of the αHL protein. Pre-expressed mCherry was added to enable fluorescence imaging. After protein expression for 2 h at 37 °C no aggregation could be observed in the absence of GUVs expressing αHL with the E3 insert. Shown is a representative image from 2 independent experiments. Scale bar: 25 µm

## Supplementary Fig. 11: GUVs expressing αHL with E3 insert do not self-aggregate.

**a**

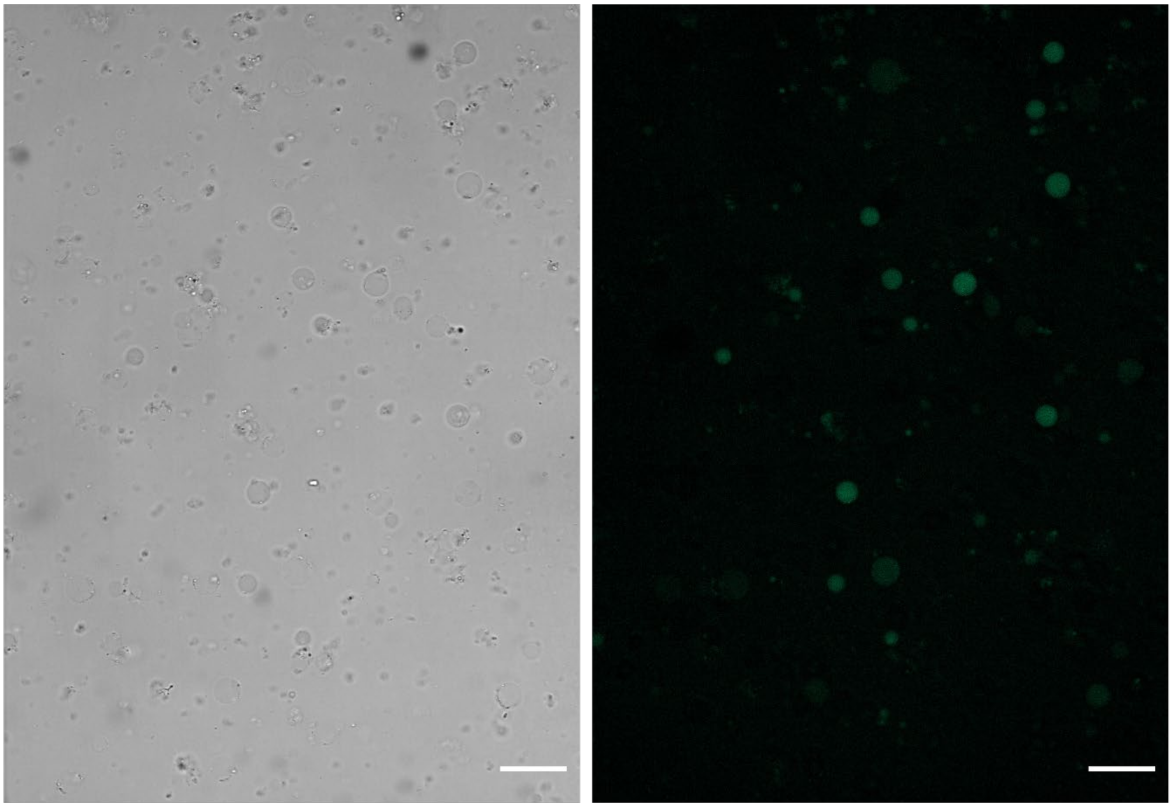

**a**, GUVs expressing αHL with the E3 insert were formed through the inverse emulsion method with a lipid composition of 60 mol% DOPC and 40 mol% cholesterol. The GUVs encapsulate the PURExpress system with a plasmid for expression of the αHL protein. Pre-expressed CFP was added to enable fluorescence imaging. After protein expression for 2 h at 37 °C no aggregation could be observed in the absence of GUVs expressing αHL with the K3 insert. Shown is a representative image from 2 independent experiments. Scale bar: 25 µm

**Supplementary Fig. 12: Hyper7 fluorescence in tissue-like structures in the absence of glucose.**

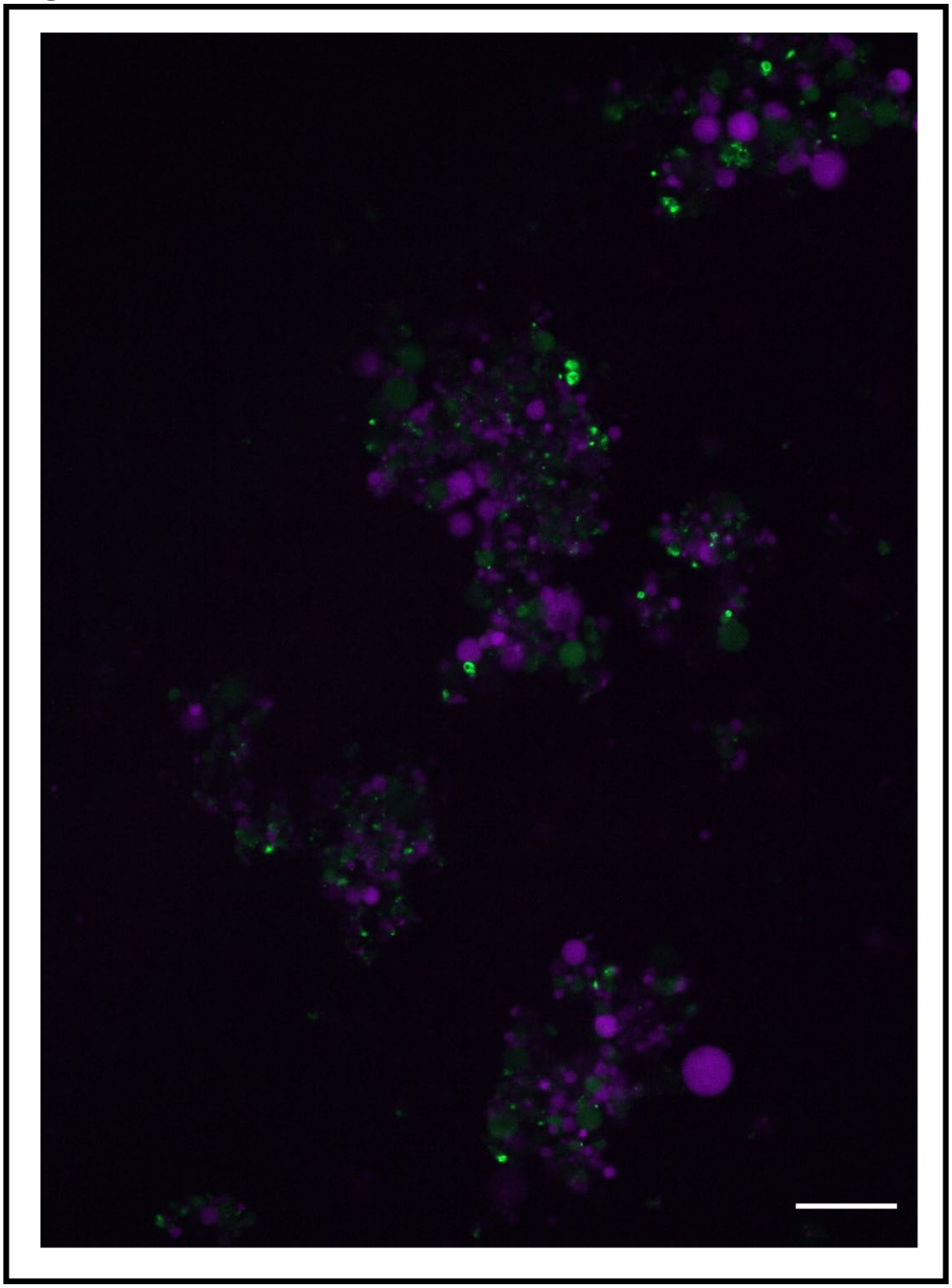

GUVs expressing αHL with either the K3 or the E3 insert were formed through the inverse emulsion method with a lipid composition of 60 mol% DOPC and 40 mol% cholesterol. For GUVs expressing αHL with the K3 insert, pre-expressed mCherry and glucose oxidase was co-encapsulated. For GUVs expressing αHL with the E3 insert, pre-expressed Hyper7 was co-encapsulated. The two populations of GUVs were mixed and incubated for 2 h at 37 °C to enable expression of the respective αHL proteins. αHL expression led to the formation of tissue-like structures. In this control experiment, no glucose was added meaning that while glucose oxidase was present in the resulting tissue like structures, no significant formation of

hydrogen peroxide was to be expected. As a result, Hyper7 fluorescence in these tissue-like structures is lower than in tissue-like structures where synthesis of hydrogen peroxide was induced by addition of glucose (Fig. 6). Shown is a representative image from 2 independent experiments.

**Supplementary Fig. 13: Hyper7 fluorescence in tissue-like structures (increased brightness in green channel)**

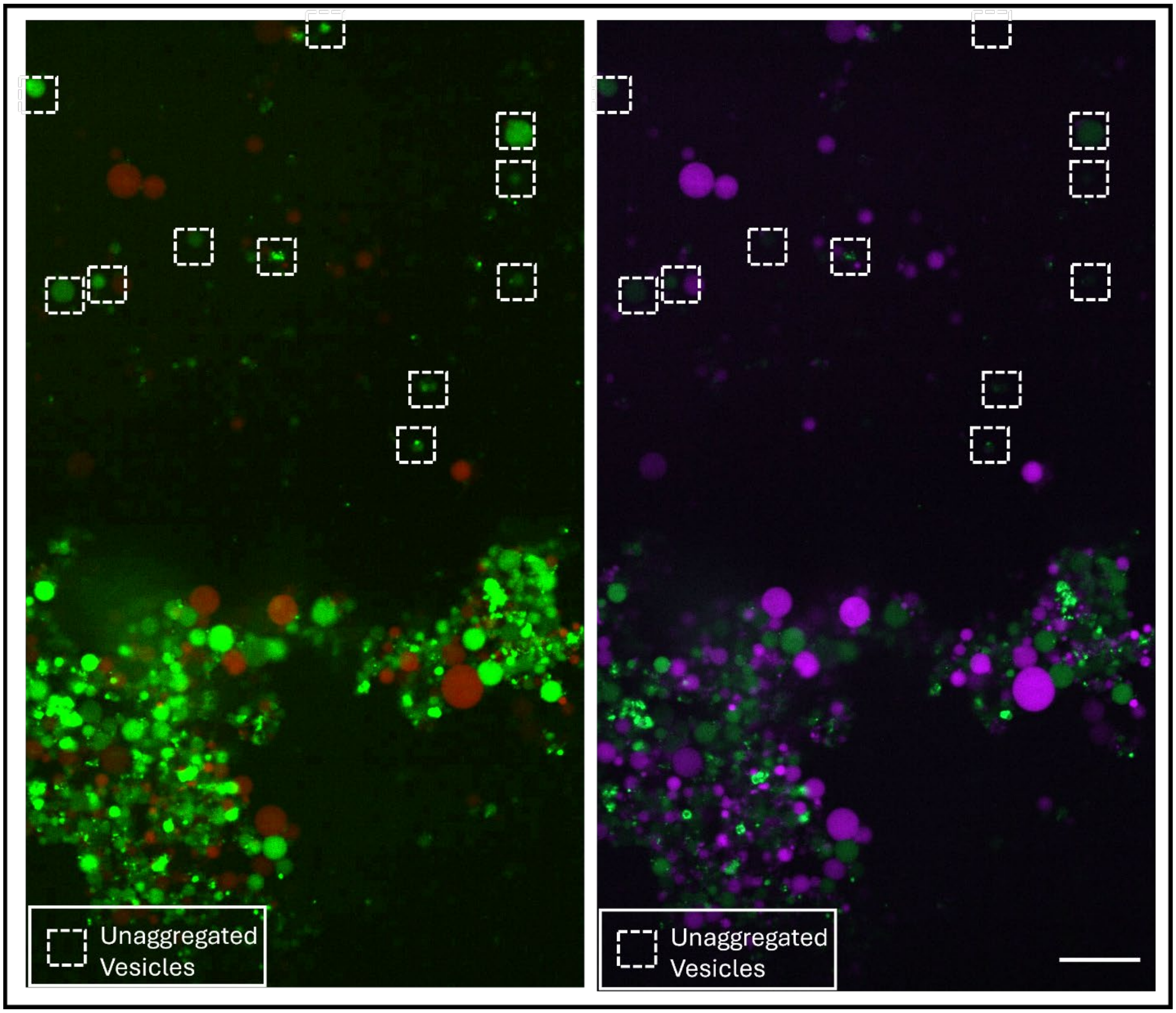


Figure 6 from main manuscript with increased brightness in the green channel to clearly visualize GUVs in the white boxes(left) next to the original figure (right).

## Supplementary Table 1: Peptide sequences which could be inserted into the αHL loop without disrupting its activity.

| Insert | Amino Acid Sequence | Length |
|---|---|---|
| **L-6XHis-L** | GGGGS HHHHHH GGGGS | 16 |
| **$L_2$-6XHis-$L_2$** | $(GGGGS)_2$ HHHHHH $(GGGGS)_2$ | 26 |
| **$L_3$-6XHis-$L_3$** | $(GGGGS)_3$ HHHHHH $(GGGGS)_3$ | 36 |
| **L-Somatostatin-L** | GGGGS AGCKNFFWKTFTSC GGGGS | 24 |
| **$L_2$-GLP1-$L_2$** | $C(GGGGS)_2$ HAEGTFTSDVSSYLEGQAAKEFIAWLVKGR $(GGGGS)_2C$ | 52 |

Inserts listed in Supplementary Table 1 were cloned into the membrane translocating loop of αHL between D128 and K131, with the inserted peptide replacing T129 and G130 as shown below. αHL proteins with these inserts proved to be fully functional, being able to self-insert into lipid bilayers and assembling into pores while translocating the respective insert across the membrane.

Full sequence of αHL with insert:

MDSDINIKTGTTDIGSNTTVKTGDLVTYDKENGMHKKVFYSFIDDKNHNKKLLVIRTKGTIAGQYRVYSEEGANKSGLA
WPSAFKVQLQLPDNEVAQISDYYPRNSIDTKEYMSTLTYGFNGNVTGDD-INSERT-KIGGLIGANVSIGHTLKYVQPDF
KTILESPTDKKVGWKVIFNNMVNQNWGPYDRDSWNPVYGNQLFMKTRNGSMKAADNFLDPNKASSLLSSGFSPDF
ATVITMDRKASKQQTNIDVIYERVRDDYQLHWTSTNWKGTNTKDKWTDRSSERYKIDWEKEEMTN

## Supplementary Table 2: Peptide inserts which lead to non-functional αHL proteins.

| Insert | Amino Acid Sequence | Length |
|---|---|---|
| **L-Sac7e-L** | GGGGS<br>MAKVRFKYKGEEKEVDTSKIKKVWRVGKMVS<br>FTYDDNGKTGRGAVSEKDAPKELMDMLARA<br>EKKK GGGGS | 75 |
| **L-SNAP-L** | GGGGS<br>MDKDCEMKRTTLDSPLGKLELSGCEQGLHRIIF<br>LGKGTSAADAVEVPAPAAVLGGPEPLMQATA<br>WLNAYFHQPEAIEEFPVPALHHPVFQQESFTR<br>QVLWKLLKVVKFGEVISYSHLAALAGNPAATA<br>AVKTALSGNPVPILIPCHRVVQGDLDVGGYEG<br>GLAVKEWLLAHEGHRLGKPGL GGGGGS | 192 |
| **L-GFP-L** | GGGGS<br>MRKGEELFTGVVPILVELDGDVNGHKFSVRGE<br>GEGDATNGKLTLKFICTTGKLPVPWPTLVTTLT<br>YGVQCFARYPDHMKQHDFFKSAMPEGYVQE<br>RTISFKDDGTYKTRAEVKFEGDTLVNRIELKGID<br>FKEDGNILGHKLEYNFNSHNVYITADKQKNGI<br>KANFKIRHNVEDGSVQLADHYQQNTPIGDGP<br>VLLPDNHYLSTQSVLSKDPNEKRDHMVLLEFV<br>TAAGITHGMDELYK<br>GGGGS | 248 |

Inserts listed in Supplementary Table 2 were cloned into the membrane translocating loop of αHL between D128 and K131, with the inserted peptide replacing T129 and G130. αHL proteins with these inserts were non-functional, meaning that they did not form pores or insert into lipid-bilayers.

## Supplementary Table 3: DNA templates used in this study.

| Name | Function | Use | Vector |
|---|---|---|---|
| pTNT-αHL-L-6XHis-L-αHL-GFP | Expression of αHL-GFP fusion with L-6XHis-L insert in BL21. | For leakage and antibody assays. | pTNT™ vector Promega |
| pTNT-αHL-$L_2$-6XHis-$L_2$-αHL-GFP | Expression of αHL-GFP fusion with $L_2$-6XHis-$L_2$ insert in BL21. | For leakage and antibody assays. | pTNT™ vector Promega |
| pTNT-αHL-$L_3$-6XHis-$L_3$-αHL-GFP | Expression of αHL-GFP fusion with $L_3$-6XHis-$L_3$ insert in BL21. | For leakage and antibody assays. | pTNT™ vector Promega |
| pTNT-αHL-L-Somatostatin-L-αHL-GFP-6XHis | Expression of αHL-GFP fusion with L-Somatostatin-L insert in BL21. | For leakage and antibody assays. | pTNT™ vector Promega |
| T7p14-αHL-L-6XHis-L-αHL | In vitro expression of αHL with L-6XHis-L insert. | For lipid bilayer recordings. | T7p14 vector myTXTL® |
| T7p14-αHL-$L_2$-GLP1-$L_2$-αHL-GFP | In vitro expression of αHL-GFP fusion with $L_2$-GLP1-$L_2$ insert. | For leakage and antibody assays. | T7p14 vector myTXTL® |
| T7p14-αHL-$L_2$-GLP1-$L_2$-αHL | In vitro expression of αHL with $L_2$-GLP1-$L_2$ insert. | For lipid channel bilayer recordings and cryo-EM sample prep. | T7p14 vector myTXTL® |
| T7p14-αHL-L-K3-L-αHL-GFP | In vitro expression of αHL-GFP fusion with L-K3-L insert. | For leakage assay. | T7p14 vector myTXTL® |
| T7p14-αHL-L-K3-L-αHL | In vitro expression of αHL with L-K3-L insert. | For use in artificial cells to encode tissue formation. | T7p14 vector myTXTL® |
| T7p14-αHL-L-E3-L-αHL-GFP | In vitro expression of αHL-GFP fusion with L-E3-L insert. | For leakage assay. | T7p14 vector myTXTL® |
| T7p14-αHL-L-E3-L-αHL | In vitro expression of αHL with L-E3-L insert. | For use in artificial cells to encode tissue formation. | T7p14 vector myTXTL® |
| T7p14-Hyper7 | In vitro expression of Hyper7. | Pre-expressed Hyper7 for encapsulation into artificial cells as a peroxide sensor. | T7p14 vector myTXTL® |

## References

1. Riener, C.K., Kada, G., Gruber, H.J. Quick measurement of protein sulfhydryls with Ellman's reagent and with 4,4'-dithiodipyridine. *Anal. Bioanal. Chem.* **373**, 266-276 (2002).

2. Bhakdi, S., Füssle, R. & Tranum-Jensen, J. Staphylococcal alpha-toxin: oligomerization of hydrophilic monomers to form amphiphilic hexamers induced through contact with deoxycholate detergent micelles. *Proc. Natl. Acad. Sci. USA* **78**, 5475–5479 (1981).

3. Walker, B., Krishnasastry, M., Zorn, L., Kasianowicz, J. & Bayley, H. Functional expression of the alpha-hemolysin of Staphylococcus aureus in intact Escherichia coli and in cell lysates. Deletion of five C-terminal amino acids selectively impairs hemolytic activity. *J. Biol. Chem.* **267**, 10902–10909 (1992).
